# Miniaturized Near-Infrared (MicroNIR) Spectrometer in Plastic Waste Sorting

**DOI:** 10.3390/ma12172740

**Published:** 2019-08-27

**Authors:** Monika Rani, Claudio Marchesi, Stefania Federici, Gianluca Rovelli, Ivano Alessandri, Irene Vassalini, Serena Ducoli, Laura Borgese, Annalisa Zacco, Fabjola Bilo, Elza Bontempi, Laura E. Depero

**Affiliations:** 1 Department of Mechanical and Industrial Engineering, University of Brescia, via Branze, 38-25123 Brescia, Italy; 2 Consorzio Interuniversitario Nazionale per la Scienza e Tecnologia dei Materiali (INSTM), 50121 Firenze, Italy; 3 ABCS Srl-Scientific Instruments & Materials Science, viale Regina Giovanna, 37-20129 Milan, Italy; 4 Department of Information Engineering, University of Brescia, via Branze, 38-25123 Brescia, Italy; 5 CNR-INO, Unit of Brescia, 25123 Brescia, Italy

**Keywords:** urban plastic waste, near-infrared (NIR) spectroscopy, chemometrics, principal component analysis (PCA), partial least squares-discriminant analysis (PLS-DA), circular economy

## Abstract

Valorisation of the urban plastic waste in high-quality recyclates is an imperative challenge in the new paradigm of the circular economy. In this scenario, a key role in the improvement of the recycling process is exerted by the optimization of waste sorting. In spite of the enormous developments achieved in the field of automated sorting systems, the quest for the reduction of cross-contamination of incompatible polymers as well as a rapid and punctual sorting of the unmatched polymers has not been sufficiently developed. In this paper, we demonstrate that a miniaturized handheld near-infrared (NIR) spectrometer can be used to successfully fingerprint and classify different plastic polymers. The investigated urban plastic waste comprised polyethylene (PE), polypropylene (PP), poly(vinyl chloride) (PVC), poly(ethylene terephthalate) (PET), and poly(styrene) (PS), collected directly in a recycling plastic waste plant, without any kind of sample washing or treatment. The application of unsupervised and supervised chemometric tools such as principal component analysis (PCA) and partial least squares-discriminant analysis (PLS-DA) on the NIR dataset resulted in a complete classification of the polymer classes. In addition, several kinds of PET (clear, blue, coloured, opaque, and boxes) were correctly classified as PET class, and PE samples with different branching degrees were properly separated.

## 1. Introduction

The huge amount of urban plastic waste and the continuous growth in human plastic consumption require a high valorisation of the collected waste in the direction of a whole-system economic sustainability. By the numbers, European plastic production reached almost 60 million tonnes in 2016, and 335 million tonnes globally (Source: PlasticsEurope Market Research Group (PEMRG)/Conversio Market & Strategy GmbH). In this framework, over 8.4 million tonnes of plastic waste were collected in order to be recycled inside and outside the EU [1]. The recycling of polymer waste has valuable environmental benefits due to the substitution of primary production, and a key role in the improvement of the recycling process is exerted by the optimization of waste sorting [2,3]. 

Generally, automated sorting processes are widespread in plastic recycling facilities [4]. These systems are usually based on vibrational spectroscopy techniques for polymer identification [5,6,7,8,9] and camera systems for optical recognition of clear and coloured products [10,11]. Other sorting technologies include UV–Vis spectroscopy [12] or mass spectroscopy (Py-GC/MS) for identification [13], hyper-spectral imaging methods [14], X-ray detection for the separation of specific containers [15], fluorescence spectroscopy for identifying halogens and heavy metals [16], and laser-induced plasma spectroscopy to detect additives [17]. This approach has increased the purity of the output plastic over the years, reaching a high percentage of recyclates in the production of secondary materials. Nevertheless, there are severe limitations of these techniques in handling mixed plastic, which requires additional sorting elsewhere and can affect the quality of the recyclate, if not properly assigned. The positive cost/benefit balance can take place only if the separated fractions of polymers match a high purity grade, fulfilling the market requirement of high recyclate quality. Therefore, a key step in post-consumer recycling is reducing the cross-contamination of incompatible polymers [10].

Besides the automated sorting of the plastic stream, manual sorting is still necessary for the full separation of the collected waste. However, manual sorting based only on plastic code recognition is inefficient and time consuming. In some cases, plastic goods can be badly crushed or have missing or unreadable codes. Manufacturers can also use different polymers for the same product, depending on marketing choices. In addition, the high turnover rate can be affected by an improperly trained or unskilled workforce.

In this scenario, the possibility of exploiting a well-established technique for polymer identification combined with a miniaturized, portable, low-cost, and real-time spectrometer for local and punctual semi-automated sorting is highly desirable. Urban plastic waste normally contains plastics such as polyethylene (PE), polypropylene (PP), poly(vinyl chloride) (PVC), poly(ethylene terephthalate) (PET), and poly(styrene) (PS). These polymers have characteristic near-infrared (NIR) spectral fingerprints that can be used for accurate sorting and separation [5]. The progress in miniaturization technologies has generated specific instrumental features that have been reported in polymer identification applications, but results have been reported only for a selection of polymers from commercial libraries, and are not fully representative of real plastic waste [18].

In the present work, we challenged a compacted and miniaturized NIR spectrometer for a rapid identification of urban plastic waste collected directly from a selection and recycling plant, without any sample treatment. We collected a large amount of plastic samples directly from the plant and we created a robust database of NIR spectra. Different chemometric tools were applied for data evaluation of all the recorded samples. Principal component analysis (PCA) for preliminary data exploration and partial least squares-discriminant analysis (PLS-DA) as supervised pattern recognition were applied to fingerprint and classify the different plastic polymers.

## 2. Materials and Methods

### 2.1. Polymer Samples Collection

Plastic samples were collected at the selection division of the recovery and recycling plant Montello SpA (Bergamo, Italy), which receives post-consumer plastic in the form of urban waste for recycling. In total, 250 samples belonging to various classes of polymer were used (see Supplementary Materials Figure S1). The polymers considered were: several kinds of PET (clear, blue, coloured, opaque, and boxes), PE with different branching degrees (low-density polyethylene, LDPE, and high-density polyethylene, HDPE), PVC, PP, and PS. The collection included bottles, containers, and packets of different shapes, sizes, and colours. The samples were flattened or partially flattened at the sampling site, and were not washed prior to testing. Black samples were not considered, due to the very low reflectance in the NIR spectral region and the low signal-to-noise ratio of NIR sensors, which hamper successful black polymer spectral acquisition [19].

### 2.2. NIR Measurements

**Figure 1:**
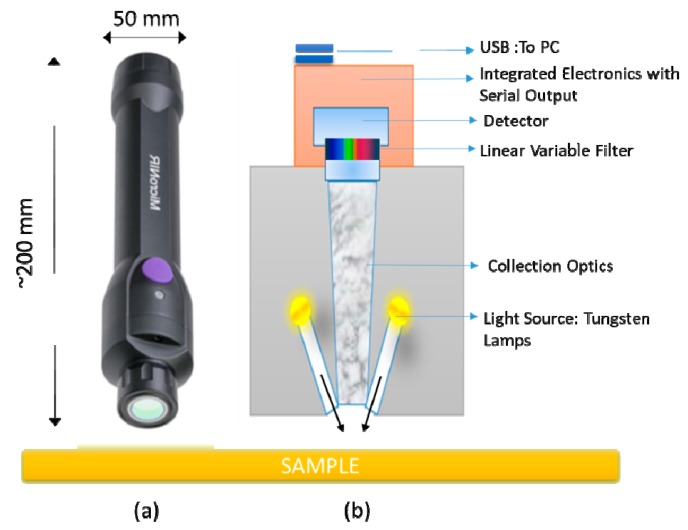
(**a**) Miniaturized near-infrared (microNIR) device and (**b**) instrument operating scheme.

Spectra acquisition was realized using MicroNIR On-site (Viavi Solutions Inc., CA, United States in reflectance mode without any sample preparation. The instrument is a miniaturized palm-sized portable spectrometer of about 250 g in weight, less than 200 mm in length, and 50 mm in diameter. This handheld spectrometer employs a linear variable filter (LVF) as dispersing element, in contrast to traditional diffraction-based spectrometers (see Figure 1 for an instrument operating scheme). The controlling parameters for spectral data collection were set at 10 ms integration time and 50 scans, resulting in a short measurement time of 0.25 s. Not less than five replicates in different positions were acquired by a point-and-shoot technique, in order to minimize effects driven by the non-uniformity of samples. A total of 1303 NIR spectra were collected. A dark and a reference scan were carried out approximately every 10 min. Data acquisition was realized through MicroNIR^TM^ Pro v3.0 software (Viavi Solutions Inc., CA, United States).

### 2.3. Signal Pretreatment

Signal correction methods are mathematical tools employed to remove additive and/or multiplicative effects on spectroscopic data, which often affect the performance of chemometric analysis [20]. These phenomena are typical of light scattering effects, which induce a photon loss (addictive effect) and an increased path length (multiplicative effect), among others. These methods are “row-wise”, and therefore the correction is made sample-by-sample. This is in contrast to mean centring and autoscaling, which are “column-wise” treatments. In most cases, the combination of the two types of method is required in order to make a satisfactory data analysis [21]. In this study, spectra were collected into a single 1307 × 125 matrix (samples × scans), and several pre-processing tools were applied to the raw data. The best result was obtained by applying Savitzky-Golay second-derivative procedure of seven data points and a second-order polynomial followed by standard normal variate (SNV). Moreover, after the preprocessing, spectra were normalized by mean centring.

### 2.4. Data Analysis

Different chemometric tools were applied in order to have a correct data evaluation of all the analysed samples. The first step consisted of an exploratory analysis by PCA aiming to explore the data structure. PCA was carried out with a total of 1303 NIR spectra for all five polymer classes. Subsequently, PLS-DA was used as supervised pattern recognition with the aim of separating the different commodities, and it was performed on a total of 1287 NIR spectra (16 spectra were removed as outliers). All calculations were performed using MATLAB (R2018a) with chemometric toolboxes [22,23].

## 3. Results and Discussion

NIR spectroscopy has the key advantage of being a rapid-response analytical tool, recording spectra with no prior manipulation and predicting physical and chemical parameters from a single spectrum [24]. In the NIR region, the absorption bands occur due to overtones or combination bands of mainly carbon–hydrogen vibrations and oxygen-hydrogen vibrations. A correct band assignment is challenging because it may be due to different combinations of fundamental vibrations. Moreover, overtone vibrations are highly overlapped [25].

**Figure 2:**
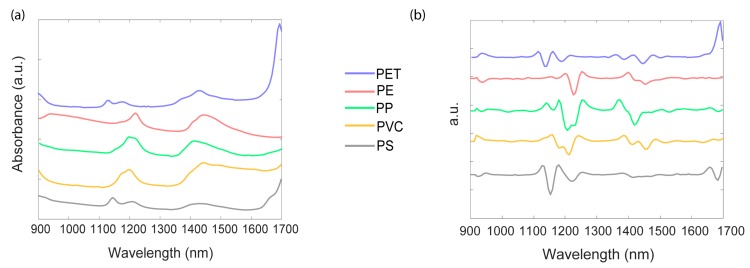
Near-infrared (NIR) spectra of five classes of plastics: (**a**) representative raw spectra of the five classes; (**b**) corresponding spectra after pre-treatment by second derivative and standard normal variate (SNV). PE: polyethylene; PET: poly(ethylene terephthalate); PP: polypropylene; PS: poly(styrene); PVC: poly(vinyl chloride).

The NIR reflectance spectra of the five polymers (i.e., PET, PP, PVC, PE, and PS) in the range 900–1700 nm, obtained by the MicroNIR On-site, are shown in Figure 2. 

**Figure A1:**
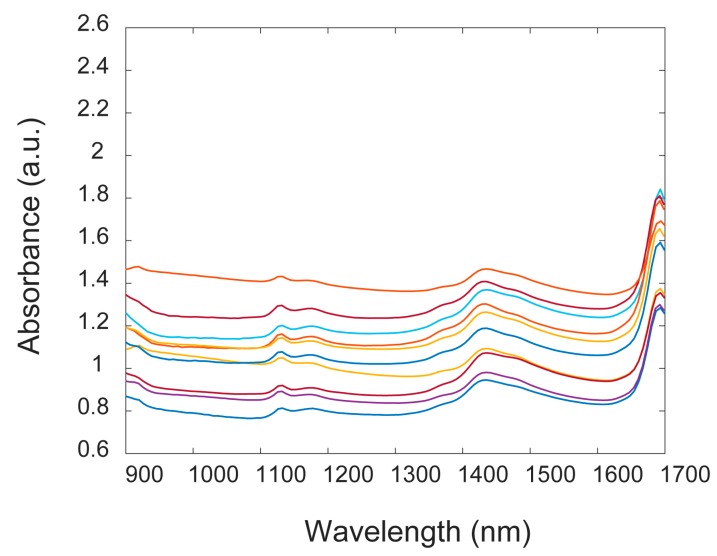
Representative NIR spectra of coloured PET samples.

**Figure A2:**
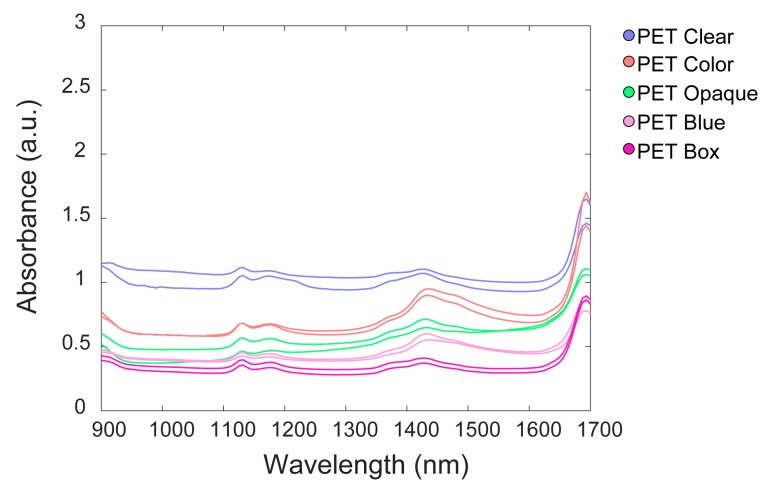
Representative NIR spectra of two random samples for each kind of PET commodity.

In the absorbance spectra (Figure 2a), the principal absorbance band for PET was found at 1660 nm due to the first overtone of C–H stretching [26], with two other small peaks around 1130 nm and 1415 nm. In the case of PVC, this main peak was shifted and was not recorded in the instrumental operating window, and only the two small peaks around 1190 nm and 1420 nm were seen [26]. The same behaviour appeared for PE and PP, assigning two bands around 1210 nm and 1430 nm for PE and around 1190 nm and 1400 nm for PP. PS exhibited a dominant band around 1675 nm and two small peaks around 1140 nm and 1205 nm [5]. Figure 2b refers to the corresponding spectra shown in Figure 2a after pre-treatment by second derivative and SNV. In general, by comparing the spectra of samples made of one type of polymer, a variation in the absorbance values was noted and this was ascribed to variations in sample thickness [5]. However, the overall form of the spectra was preserved, concluding that dust or liquid contaminations randomly characterizing each sample did not affect the quality of NIR spectra (see Appendix A, Figure A1) [27]. In addition, NIR spectra of samples of different colours did not display considerable differences (Figure A1), meaning that the colorants present in the plastic resin matrix did not significantly influence the NIR spectra. Moreover, no differences in band positions were found when studying the spectra of several kinds of PET (clear, coloured, opaque, box, and blue) (see Appendix A, Figure A2), implying that the spectra can be successfully exploited to discriminate PET samples, despite their differences. For this reason, hereinafter, only one PET class is considered.

### 3.1. Principal Component Analysis

**Figure 3:**
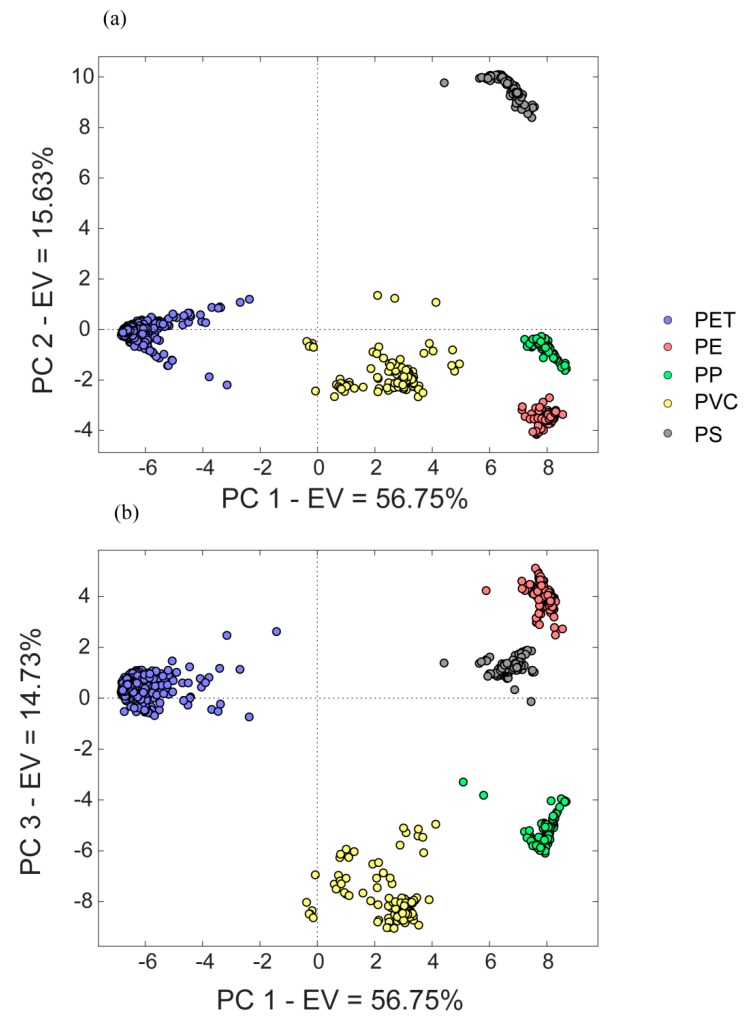
Results of principal components analysis carried out with spectral data of the different commodities. (**a**) The score plot of the first two components is shown, as well as (**b**) the score plot of PC1 vs. PC3 EV: explained variance.

Principal component analysis is a very powerful chemometric tool for analysing data structure [28]. The aim of PCA is to extract the information encoded in a certain number of variables into a smaller set of new orthogonal variables called principal components. PCA calculation was performed over the entire spectral region and using all collected spectra. The first three components accounted for 87.11% of the total variability (PC1 56.75%, PC2 15.63%, and PC3 14.73%). Figure 3 displays the score plots of PC1 vs. PC2 (Figure 3a) and PC1 vs. PC3 (Figure 3b). In the score plot displayed in Figure 3a, a clear separation between the five commodities can be seen. Indeed, in the new space determined by the first two components, polymers formed very tight and homogeneous clusters. Along the first component, a clear differentiation between PET, PVC, PP, and PE was highlighted, while the second component allowed the differentiation of PS from the other polymers, and PE from PP. On the other hand, Figure 3b shows the score plot of PC1 vs. PC3. In this case PET was differentiated from the other polymers along PC1, while PC3 distinguished between the remaining sets of polymers. 

**Figure 4:**
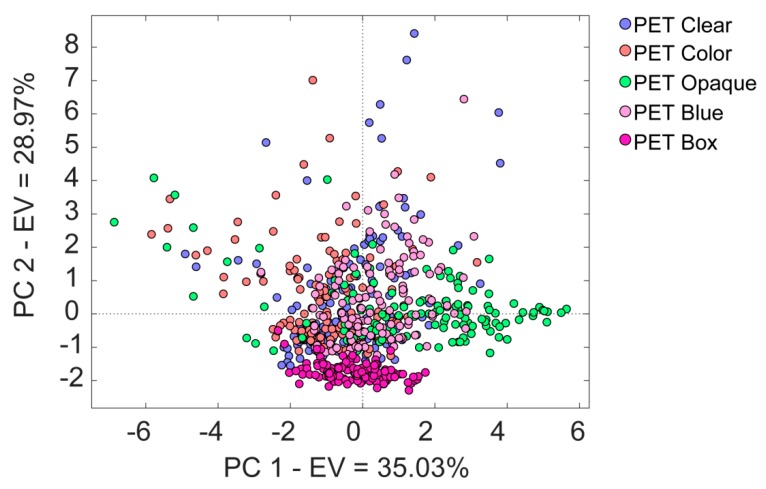
Principal component analysis (PCA) analysis of several kinds of poly(ethylene terephthalate) (PET) samples for a total of 659 spectra considered. EV: explained variance.

Furthermore, the results of PCA applied to the restricted dataset of PET samples showed no separation among the five kinds of PET samples (Figure 4), confirming the interpretation of the band assignment of NIR spectra.

### 3.2. HDPE versus LDPE

**Figure 5:**
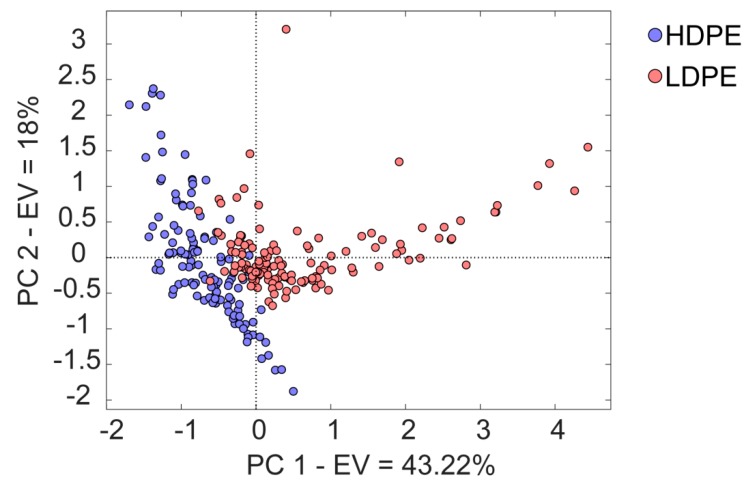
Results of principal components analysis carried out with spectral data of polyethylene (PE) class polymers. Score plot of the first two components is shown.

The discrimination between HDPE and LDPE is an important issue in terms of plastic recycling [3]. PE is largely used in the food packaging sector, thanks to its remarkable properties in terms of mechanical and optical performances, water-vapor-resistance, and heat-sealing attitude. The amount of side branches that are attached to the main PE polymer chain determines the morphological properties, which in turn affects optical, physical, and thermal properties [29,30]. NIR spectroscopy successfully discriminates between HDPE and LDPE [31,32], even if severe limitations can occur depending on the characteristics of the measuring instruments [27]. In the sampling campaign, we collected 25 samples of HDPE and 25 samples of LDPE. Raw NIR spectra did not show any significant feature to discriminate the two plastics, nor did a clear separation in the score plots resulting from the previous PCA analysis (Figure 3). However, by limiting the PCA analysis to the PE samples, a separation between HDPE and LDPE was obtained; the first two components explicated 61.22% of the total variability (PC1 43.22%, PC2 18.00%), confirming the successful application of NIR spectroscopy in discriminating these two polymers. Figure 5 displays the score plots of PC1 vs. PC2. 

### 3.3. Partial Least Squares-Discriminant Analysis

After the exploratory PCA analysis, a supervised classification tool was applied in order to distinguish the different plastic classes. In PLS-DA, the well-known PLS regression algorithm is modified with a classification goal. The response variable is categorical, reflecting the belonging class of the statistical units. PLS-DA returns the prediction in a vector of size equal to the number of classes in the predictor variables, with values ranging from 0 to 1 [33].

Validation tools are very useful methods in chemometrics, and they are used to verify the capability of the model prediction. Before the model calibration, data were split into training and test sets containing 901 and 386 samples, respectively. The training set was used to calibrate the model both in fitting and in cross-validation, while the test set was employed only at the end of the procedure in order to evaluate the true predictive capability of the calibrated model [22].

**Figure 6:**
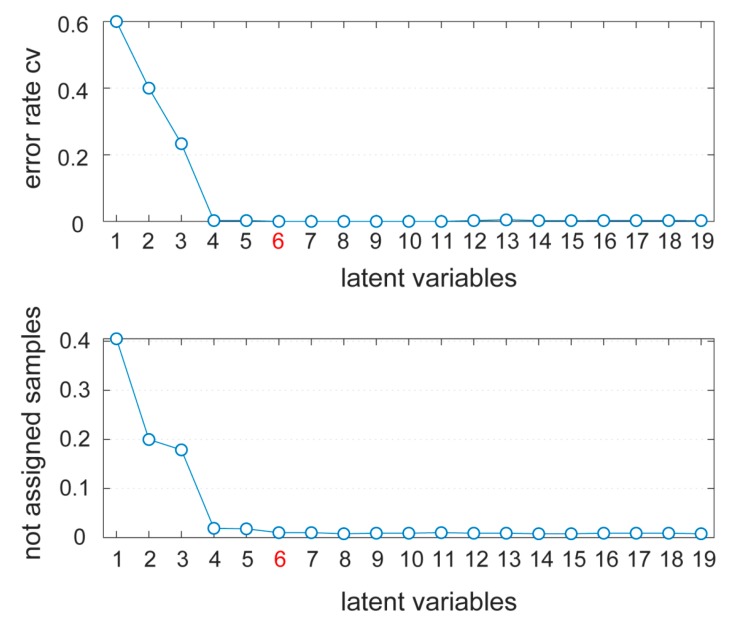
Error rate (**a**) and not assigned samples (**b**) as a function of latent variables calculated in the partial least squares-discriminant analysis (PLS-DA) model. Six latent variables (LVs) was the optimal number, marked in red, with 0.0099% of not assigned samples.

During the calibration, the selection of the optimal number of latent variables (LVs) is a crucial point, which is performed based on the cross-validation procedure [22]. This should allow the optimization of the complexity of the multivariate model according to the predictive capacity of the model itself. Cross-validation is generally carried out by dividing the training set into different cross-validation groups, and during each round one group is removed from the training set. Over the rounds, the model is calibrated on the remaining training samples and then used to predict samples of the cross-validation group. The cross-validation procedure was based on a Venetian blinds approach with 10 groups. In the Venetian blinds procedure, the calibration group and the cross-validation group are selected by choosing every *n*-th sample from the dataset starting from the first one [22]. The selection of the number of cross-validation groups was set to 10, as a reasonable number in order to avoid the so-called “overfitting” (i.e., the overestimation of the predictive capability of the model). Figure 6 shows the error rate (Figure 6a) and the degree of not assigned samples as a function of the number of latent variables (Figure 6b). The optimal number of LVs was set to 6 (explaining 94% of the total variability and 0.0099% of not assigned samples), which was associated with the minimum error rate and, simultaneously, with the minimum percentage of not assigned samples. Four LVs reached the same error rate, but 0.019% of not assigned samples.

After the determination of the optimal number of LVs, the calculation of the PLS-DA model was performed selecting six latent variables and 10 cross-validation groups for internal validation. In Table 1, the two confusion matrices achieved in fitting and in cross-validation were presented. Both in fitting and in cross-validation, the performance of the model was very high, proved by the correlation between observed and predicted classes. All the investigated samples were correctly designated, and only four samples in fitting and five in cross-validation were not assigned. 

The performance of the classification model could be also evaluated by considering the classification parameters derived from the confusion matrix: sensitivity (Sn), specificity (Sp), and non-error rate (NER). These parameters represent the ability of the model to correctly identify the samples of the given class, the ability of a classifier to reject the samples of other classes, and the average of the class sensitivities, respectively [34]. Table 2 collects the parameters of the PLS-DA model. In both fitting and cross-validation, NER, Sn, and Sp were equal to 1, meaning that 100% of the polymers were correctly classified.

Furthermore, we analysed the confusion matrix and the classification parameters for the test set, used as an additional trial to validate the model. Table 3 shows the confusion matrix derived from the test set; again, 100% of the samples were classified, and only four samples were not assigned. Table 4 summarizes the classification parameters obtained from the confusion matrix, with the NER, Sn, and Sp of each class equal to 1.

In the case of the restricted data set composed of HDPE and LDPE spectra, the optimal number of LVs was set to six, assigning 90% of total variability (see Supplementary Materials, Figure S2). With respect to the analysis of the polymer classes, the degree of not assigned samples as a function of the number of latent variables was equal to zero, meaning that all the samples were assigned to a class. After the determination of the optimal number of LVs, the calculation of the PLS-DA model was performed, selecting six latent variables and 10 cross-validation groups for internal validation. Both in fitting and in cross-validation, all the investigated samples were correctly designated (see Table S1 in the Supplementary Materials for the two confusion matrices achieved in fitting and in cross-validation).

## 4. Conclusions

In the present study, the use of a handheld NIR spectrometer combined with robust chemometrics tools to fingerprint different urban plastic waste collected directly from a recycling plant was presented. Plastic samples included clear, blue, coloured, opaque, and boxes of PET, PE with different branching degrees (HDPE and LDPE), PP, PVC, and PS. In a first step, PCA was applied to NIR spectra of all polymer classes, and polymers were correctly separated in clusters. Moreover, by limiting the analysis on PE samples, HDPE and LDPE were correctly discriminated. 

In addition, by comparing samples of different colours, no differences in band positions were found, suggesting that colorants present in the plastic resin matrix do not significantly influence NIR spectra. Moreover, even if samples were not washed or treated prior to the analysis, NIR spectra were not affected by dust contamination or liquid residuals, meaning a successful application directly to the urban plastic waste.

After the exploratory PCA analysis, PLS-DA was applied. Before the model calibration, data were split into a training and test set, using the training set to calibrate the model. In both fitting and cross-validation, the performance of the model was very high, proved by the correlation between observed and predicted classes. All the analysed samples were also correctly designated in the case of the restricted data set composed of HDPE and LDPE spectra.

The results show promising outcomes in order to improve the reliability and efficiency of the manual sorting of plastic waste, increasing the volume of recycled plastic and the management of multi-component polymer types.

## Figures and Tables

**Table 1:** Confusion matrices obtained from the PLS-DA model, both in fitting and in cross-validation (based on Venetian blinds with 10 groups). The “Not Assigned” column contains samples which were not assigned to any of the considered classes.

Experimental Class	Calculated Class					
	PET	PE	PP	PVC	PS	Not Assigned
Fitting	-	-	-	-	-	-
PET	459	0	0	0	0	2
PE	0	181	0	0	0	1
PP	0	0	82	0	0	0
PVC	0	0	0	84	0	1
PS	0	0	0	0	91	0
Cross-Validation	-	-	-	-	-	-
PET	460	0	0	0	0	1
PE	0	181	0	0	0	1
PP	0	0	82	0	0	0
PVC	0	0	0	82	0	3
PS	0	0	0	0	91	0

**Table 2:** Classification parameters (non-error rate (NER), class sensitivity (Sn), and specificity (Sp)) calculated in fitting and in cross-validation.

-	-	PET		PE		PP		PVC		PS	
	NER	Sn	Sp	Sn	Sp	Sn	Sp	Sn	Sp	Sn	Sp
Fitting	1	1	1	1	1	1	1	1	1	1	1
Cross-validation	1	1	1	1	1	1	1	1	1	1	1

**Table 3:** Confusion matrix obtained from the PLS-DA model fitted on the validation set. The “Not Assigned” column contains samples which were not assigned to any of the considered classes.

Experimental Class	Calculated Class					
	PET	PE	PP	PVC	PS	Not Assigned
PET	197	0	0	0	0	1
PE	0	78	0	0	0	1
PP	0	0	33	0	0	1
PVC	0	0	0	35	0	1
PS	0	0	0	0	39	0

**Table 4:** Classification parameters (non-error rate (NER), class sensitivity (Sn), and specificity (Sp)) calculated on the validation set.

-	-	PET		PE		PP		PVC		PS	
	NER	Sn	Sp	Sn	Sp	Sn	Sp	Sn	Sp	Sn	Sp
Test	1	1	1	1	1	1	1	1	1	1	1

## Supplementary Materials

The following are available online at https://www.mdpi.com/1996-1944/12/17/2740/s1. Figure S1: Systematic organization of the collected urban plastic waste for laboratory measurements, Figure S2: Error rate as a function of latent variables calculated in the PLS-DA model for HDPE and LDPE classification. Six LVs was the optimal number, Table S1: Confusion matrices obtained from PLS-DA model, both in fitting and in cross-validation (based on Venetian blinds with 10 groups) restricted to the PE class.

## Appendix A

### Appendix A.1. Evaluation of Sample Contaminations and Colour Influence

Figure A1 shows a representative collection of NIR spectra of coloured PET samples. The coloured PET batch was chosen as a demonstrator set to compare samples with random contaminations and to evaluate the presence of colorants. A total of 136 spectra were collected (25 samples with at least 5 replicates in different positions for each sample). The samples obtained from the recycling plant were flattened or partially flattened directly on site and were not washed prior the testing. Liquid residuals, pieces of labels, and environmental dust were largely present on the surface and inside the samples. Nevertheless, NIR spectra showed only a variation in the total absorbance values, attributable to variations in sample thickness, according to Lambert–Beer law [35]. Moreover, no differences in band positions were found despite the different colours of the commodities.

### Appendix A.2. PET Samples

Several kinds of PET were considered. In particular, clear, coloured, opaque, blue, and box samples of PET were collected at the recycling plant (25 samples for each kind). In order to investigate the NIR contribution of the different kinds, we acquired at least five replicates for each sample (for a total of 659 spectra) to make spectra comparison and multivariate analysis. Figure A2 shows representative NIR spectra of a restriction of two random samples for each kind, in order to better visualize the spectra in the plot. The overall form of the spectra was preserved and no significant differences are noted.
