# Supplementary material for: Miniaturized Near-Infrared (MicroNIR) Spectrometer in Plastic Waste Sorting
Source: Materials (Basel). 2019 Aug 27;12(17):2740. doi: 10.3390/ma12172740 (PMC6747759; doi:10.3390/ma12172740)
Supplement: Supplementary file 1 [file materials-12-02740-s001.pdf]

materials

Article

Miniaturized Near-Infrared (MicroNIR) Spectrometer in Plastic Waste Sorting

Monika Rani [1,2,‡], Claudio Marchesi [1,2,‡], Stefania Federici [1,2,*], Gianluca Rovelli [3], Ivano Alessandri [2,4,5], Irene Vassalini [2,4,5], Serena Ducoli [2,4], Laura Borgese [1,2], Annalisa Zacco [1,2], Fabjola Bilo [1,2], Elza Bontempi [1,2] and Laura E. Depero [1,2]

1 Department of Mechanical and Industrial Engineering, University of Brescia, via Branze, 38-25123, Brescia, Italy
2 Consorzio Interuniversitario Nazionale per la Scienza e Tecnologia dei Materiali (INSTM), 50121 Firenze, Italy
3 ABCS Srl-Scientific Instruments & Materials Science, viale Regina Giovanna, 37-20129 Milan, Italy
4 Department of Information Engineering, University of Brescia, via Branze, 38–25123, Brescia, Italy
5 CNR-INO, Unit of Brescia, 25123 Brescia, Italy
‡ these authors are equally contributed.
* Correspondence: stefania.federici@unibs.it

Supplementary Materials

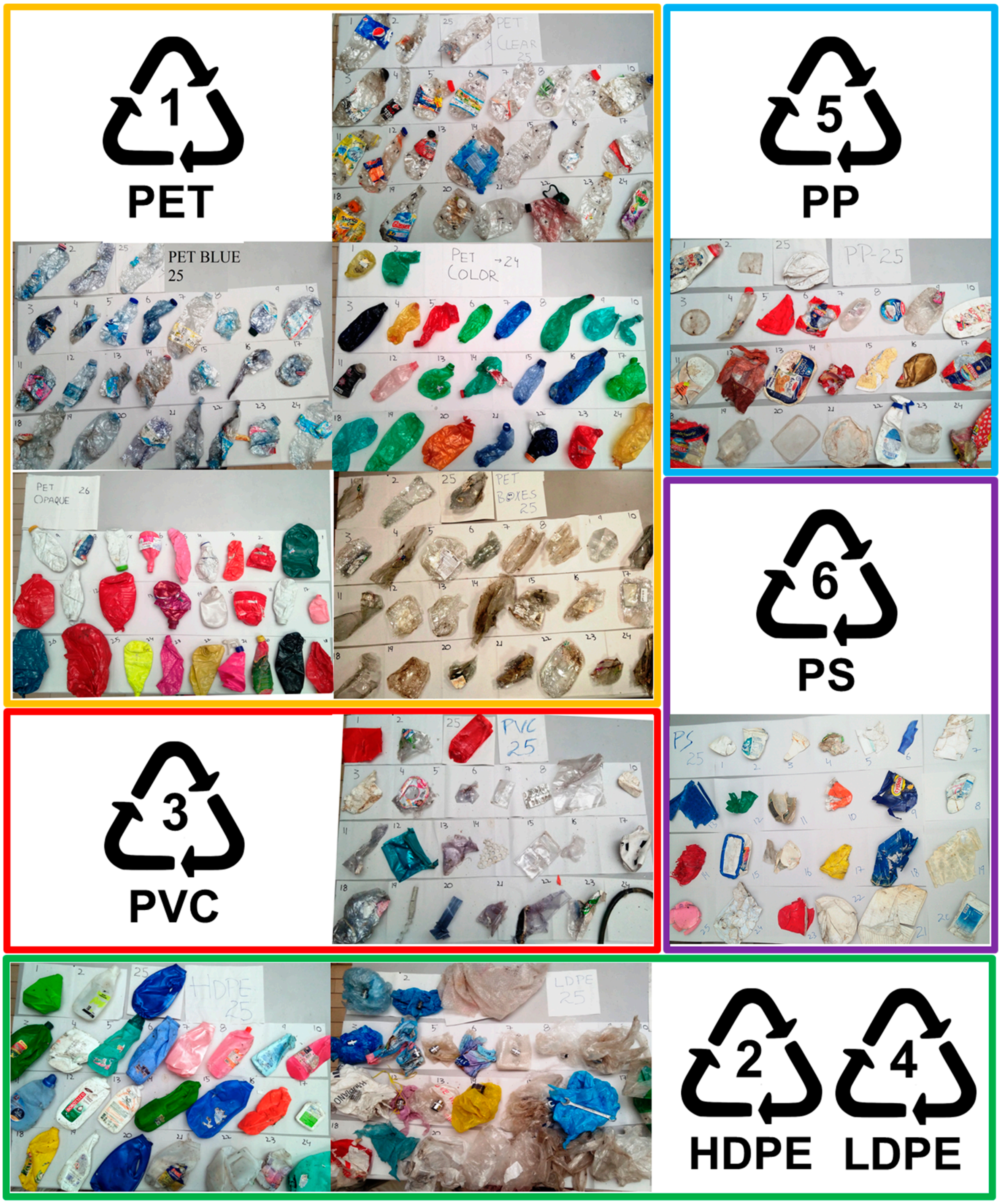

Figure S1. Systematic organization of the collected urban plastic waste for laboratory measurements.

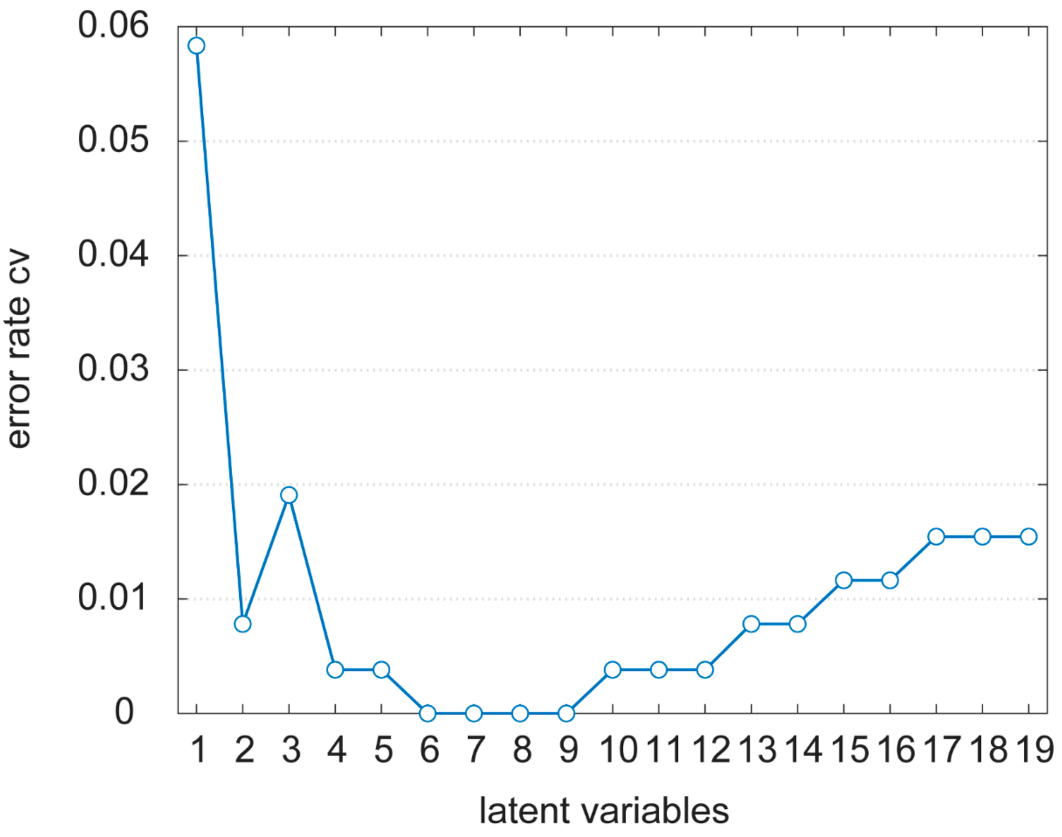

Figure S2. Error rate as a function of latent variables calculated in the PLS-DA model for HDPE and LDPE classification. 6 LVs is the optimal number.

Table S1. Confusion matrices obtained from PLS-DA model, both in fitting and in cross-validation (based on venetian blinds with 10 groups) restricted to PE class.

Experimental Class	Calculated Class		
	HDPE	LDPE	Not Assigned
Fitting	-	-	-
HDPE	125	0	0
LDPE	0	131	0
Cross-Validation	-	-	-
HDPE	125	0	0
LDPE	0	131	0
